# Immunization against Rumen Methanogenesis by Vaccination with a New Recombinant Protein

**DOI:** 10.1371/journal.pone.0140086

**Published:** 2015-10-07

**Authors:** Litai Zhang, Xiaofeng Huang, Bai Xue, Quanhui Peng, Zhisheng Wang, Tianhai Yan, Lizhi Wang

**Affiliations:** 1 Institute of animal nutrition, Sichuan Agricultural University, Yaan, Sichuan, China; 2 Agri-Food and Biosciences Institute, Hillsborough, United Kingdom; University of Queensland, AUSTRALIA

## Abstract

Vaccination through recombinant proteins against rumen methanogenesis provides a mitigation approach to reduce enteric methane (CH_4_) emissions in ruminants. The objective of present study was to evaluate the *in vivo* efficacy of a new vaccine candidate protein (EhaF) on methanogenesis and microbial population in the rumen of goats. We amplified the gene mru 1407 encoding protein EhaF using fresh rumen fluid samples of mature goats and successfully expressed recombinant protein (EhaF) in *Escherichia coli* Rosetta. This product was evaluated using 12 mature goats with half for control and other half injected with 400ug/goat the purified recombinant protein in day 1 and two subsequent booster immunizations in day 35 and 49. All measurements were undertaken from 63 to 68 days after the initial vaccination, with CH_4_ emissions determined using respiration calorimeter chambers. The results showed that the vaccination caused intensive immune responses in serum and saliva, although it had no significant effect on total enteric CH_4_ emissions and methanogen population in the rumen, when compared with the control goats. However, the vaccination altered the composition of rumen bacteria, especially the abundance of main phylum Firmicutes and genus *Prevotella*. The results indicate that protein EhaF might not be an effective vaccine to reduce enteric CH4 emissions but our vaccine have potential to influence the rumen ecosystem of goats.

## Introduction

Methane (CH_4_) is a long-lived greenhouse gas (GHG) and over 25 times more potent than carbon dioxide (CO_2_) in its global warming potential [1]. Globally, CH_4_ emissions from the agricultural sources account for about 40% of the total human-induced CH_4_ [2] and 25% of which from the enteric fermentation in ruminants [3]. In addition, CH_4_ emissions represent a loss of about 5 to 7% of dietary energy and are about 16 to 26 g/kg of DM intake [4]. Therefore, reducing CH_4_ emissions from ruminants not only benefits for environment, but also improves the ruminant production efficiency.

A range of strategies have been investigated recently to mitigate CH_4_ emissions from ruminants [5–7], including the development of vaccines that specifically target methanogens in the rumen. Previous studies have shown that there were immunogenic fractions on the surface of methanogens and antibodies generated against that fractions could curtail the activity of methanogens in ruminants [8–10]. However, most of the vaccines against methanogens failed to produce the promising results, due to low efficacy, or poor cross-reactivity in various methanogens [11, 12].

The development of reverse vaccinology has given new and convenient solutions to identify suitable, high-performance and conserved vaccines [13] and there have been several successful applications of this technology to identify vaccine candidates against bacterial pathogens [14–16]. Sequencing the genome of *Methanobrevibacter ruminantium* M1 has opened new frontiers and provided data for identifying conserved vaccine targets among all methanogens in the rumen via reverse vaccinology. Several gene targets encoded M1 adhesin-like proteins have been identified to inhibit CH_4_ emissions in M1 and immune sera produced by small peptides synthesized to correspond to these proteins are shown to bind specifically to immobilized M1 cells [17].

Leahy et al. identified 47 ORFs of potential vaccine targets through bioinformation technology, which have high degree of conservation among methanogens and are suitable for cloning and heterologous expression studies [17]. The mru1407 gene (GenBank Accession NC_013790) is one of them and encodes protein EhaF (energy-converting hydrogenase A subunit F), which plays an essential anaplerotic role in hydrogenotrophic methanogenesis and is essential for growth of methanogens [18]. To date, there has been no application to use this protein as vaccine against rumen methanogenesis.

Therefore, the objectives of the present study were to develop the above mentioned protein (EhaF) in *E*.*coli* and to evaluate its effects as a vaccine candidate on the methanogenesis, microbial population and enteric CH_4_ emissions in mature goats.

## Materials and Methods

### Gene cloning, expression and purification

The mature Boer goats used in the present study were reared in the research farm of Sichuan Agricultural University, Ya’an, Sichuan, China. The experiment procedure was approved by the Animal Care and Ethics Committee of Sichuan Agricultural University (Permit Number: DKY-S20112806). The fresh rumen contents were obtained from three 18-month old and healthy Boer goat (33.3±0.4 kg) immediately after euthanasia by intravenous injection of 3 mg/kg BW of chlorpromazine hydrochloride (Shanghai Harvest Pharmaceutical Co. Ltd. Shanghai, China). The three samples were strained respectively through 4 layers of sterile cheese cloth. A liquid sample with an equal volume was taken from each goat, the three liquid samples were then completely mixed, and afterwards a composite supernatant sample was collected for total RNA extraction. Total RNA was extracted from the supernatant using TRIZOL (TaKara, Japan) and products were reverse-transcribed using PrimeScript RT reagent kit with gDNAeraser (TaKara, Japan) as described before [19]. The gene mru 1407 was amplified from the cDNA by PCR using forward primer: 5’-AAAACTCTGAA-GGAGGCAAAT–3’ and reverse primer: 5’-AGACGGTTAAGTTGATCT–3’ which was designed according to the sequence of mru 1407 and mru 1408 (GenBank Accession NC_013790). The PCR procedure comprised an initial step of 5 min at 95°C, a second step of 35 cycles including 30 s at 95°C, 30 s at 58°C and 90 s at 72°C, and a final extension step of 10 min at 72°C. The product was ligated with pMD18-T (Takara, Japan) to construct recombinant plasmid for transformation of DH5ɑ competent cells (Tiangen, China). The positive recombinant plasmid was sequenced and the sequence of mru 1407 was submitted to Genbank (Accession KP453861). The forward primer 5’-TATCGGATCCATGCC-TAAAATTGCAAA–3’ and reverse primer 5’-CCGCAAGCTTAC CTGAACTCCTTTTTAG–3’ with *Bam*H I and *Hin*d III restriction site (underlined) were used to amplify mru1407 from that recombinant plasmid by PCR. The PCR program consisted of a single cycle of denaturation at 95°C for 5 min, 30 cycles at 95°C for 30 s, 56°C for 30 s, and 72°C for 90 s, followed by a single cycle of extension at 72°C for 10 min. Afterwards, the products were cloned into the pET-30a (+) (Novagen, Germany) expression vector and the plasmid pET-30a (+)-mru 1407 obtained was transformed into host bacteria *E*. *coli* Rosetta (DE3) (Novagen, Germany) with the empty pET-30a (+) for control. The expression host was cultured for 16 h in TB medium with 0.5% (v/v) glycerol, 0.05% (w/v) glucose and 0.2% (w/v) ɑ-lactose at 30°C with shaking at 250 rpm. The bacteria were harvested by centrifugation and stored as a frozen pellet at –70°C. The pellet was then used by adding the Lysis buffer (50mM Tris-HCl, PH 8.0, 500mM NaCl, 1mg/ml lysozyme, 0.1% Triton X–100) and stirred slowly at room temperature for 10 min, then broken on ice by ultrasonic fragmentation (Misonix, USA) for 8 min. The supernatant was collected by centrifugation, then flowed through Ni-NTA Agarose (Qiagen, Germany) and washed twice with Wash Buffer (50mM Tris-HCl, PH 8.0, 500mM NaCl, 20mM imidazole). The target protein was eluted with Elution Buffer (50mM Tris-HCl, PH 8.0, 500mM NaCl, 500mM imidazole). The protein was concentrated using an Amicon Ultra–4 centrifugal filter (Millipore, USA) and monitored via SDS-PAGE. The concentration of this protein was determined according to the Bradford color-reaction assay with bovine serum albumin as a standard [20].

### Mass spectrometric analysis of recombinant protein

The purified protein was run on the SDS-PAGE and a 1.5-mm diameter gel plug was cut from band of interest. The sample was destined with the solution containing 50% acetonitrile (ACN)/50 mM NH_4_HCO_3_. The gel slice was dewatered by ACN and finally dried in N_2_. Trypsin (trypsin 12.5 ng/μl and 20mM NH_4_HCO_3_) was added to the sample and incubated at 37°C overnight. The tryptic peptides were extracted twice with the extract solution (5% formic acid in 50% acetonitrile). The matrix solution (5mg/ml α-cyano-4-hydroxy-cinnamic acid diluted in 0.1% TFA, 50% ACN) was pipetted to dissolve it. Then the peptide solution was subjected to AB SCIEX 5800 TOF/TOF analysis. The MS data were searched by GPS Explorer (V3.6) with the search engine MASCOT (V2.3).

### Immunization protocol

Twelve 18-month old Boer goats, reared in the research farm of Sichuan Agricultural University and offered the diet as described previously, were allocated into two groups (6/group) according to their age and live weight. All goats were offered a complete diet ad libitum, with 30% concentrate and 70% forage (50% alfalfa hay and 50% rice straw) (DM basis). After two weeks for the adaption, each animal in the vaccination group was injected intradermally with 1ml Elution Buffer containing 400ug purified protein EhaF emulsified with Freund’s complete adjuvant (FCA) (Sigma, USA) at 8 sites in the neck area. Two boosters in Freund’s incomplete adjuvant (FIA) (Sigma, USA) were given afterwards in 35 and 49 days after the initial injection. Animals in the control group were each injected with 1ml Elution Buffer mixed with FCA or FIA at the same time schedule with those in the treatment group. Animals were closely monitored during the whole feeding period and all procedures for animal care and injection were undertaken following the guidelines set up by the Animal Care and Ethics Committee of Sichuan Agricultural University (Permit Number: DKY-S20112806). No health problems of animals were observed during the feeding period.

Serum and Saliva samples from each goat were taken on day 63 and stored at -20°C until required. The saliva was collected using a swab placed in the mouth which was then put into the Salivette (Sarstedt, Numbrecht, Germany). The latter was centrifuged for 2 min at 1000g to yield a clear saliva sample.

### Methane emission measurements

After collecting serum and saliva samples on day 63, the animals were transferred to indirect open-circuit respiration chambers. The animals were housed in the chambers for four days with feed intake and CH_4_ emissions measured during the final three days. All procedures, analytical methods, and calculations used in the calorimetric experiment were as reported previous [21]. Immediately after the completion of chamber measurements, a rumen content sample was collected from each goat before feeding in the morning using a stomach tube attached to an electric pump as described previously [22]. The samples were then strained through four layers of sterile cheese cloth and the rumen fluid was immediately stored in liquid nitrogen for latter DNA extraction and ELISA (the enzyme-linked immunosorbent assay).

### ELISA

An indirect ELISA was used to measure antibodies presented in the samples of serum, saliva and rumen fluid. Before the measurements, wells of microplates (Maxisorp, Nunc, Danmark) were coated with 2μg/ml solution of purified recombinant protein in carbonate buffer (PH 9.6) for 12 h at 4°C. After three washes with PBS containing 0.05% w/v Tween–20 (PBST), the plates were blocked with 5% bovine serum albumin (BSA) in PBS (200μl per well) for 1 h at 37°C, followed by washing with PBST. Samples of serum (initial dilution 1:500), saliva (undiluted) or rumen fluid (initial undiluted) were added to appropriate wells at 100μl per well, and the plates were incubated for 2 h at 37°C. After washing as above, Anti-goat IgG (H+L)-Peroxidase antibody produced in donkey (Sigma, USA) diluted at 1:10000 was added to each well and the plates were again incubated at 37°C for 1 h. Plates were washed 3 times as above, each well was added 50μl of 3, 3', 5, 5'-Tetramethylbenzidine (TMB) (Sigma, USA) and incubated at room temperature in the dark for 20 min. The color reaction was stopped by adding 100μl 2M H_2_SO_4_/well and optical densities at 492 nm (OD_492_) were measured. Results were expressed in titres, defined as the reciprocal of the dilution of serum, saliva or rumen fluid that gave half the maximal optical density [10].

### DNA extraction, sequencing and data analyses

The total DNA of rumen samples was extracted with TIANamp Bacteria DNA Kit (Tiangen, China) according to the instructions provided by the manufacturer. The purified DNA was amplified in triplicate by PCR, using a universal bacteria archaea 16S rRNA gene (variable region V4) forward 515F (5′-GTGYCAGCMGCCGCGGTAA–3′) and reverse 806R (5′-GGACTAC HVGGGTWTCTAAT–3′) primer pairs [23]. The PCR mixtures were made with 4μl of FastPfu Polymerase (Transgen, Bio Inc., China), 4μl of 5×FastPfu Buffer, 5μM of each forward and reverse primers, 2μl 2.5mM dNTP, 10ng DNA template and ddH_2_O in a total volume of 20μl. The PCR program was started at 94°C for 2 min, then performed for 30 cycles (95°C for 30 seconds, 55°C for 30 seconds, 72°C for 45 seconds), following by an extension step at 72°C for 10 min. The products of PCR were subjected to sequencing by using a MiSeq PE250 platform (Illumina, USA). The data of sequencing were analyzed mostly using QIIME pipeline (version 1.8.0) [24]. All the sequences were assigned into each sample based on the barcode and performed quality filtering (100bp<sequence length<300bp, Phred quality score>20). The UCLUST was used to cluster the sequences into OTUs (Operational Taxonomic Units) and choose the representative sequence of each OTU at 97% similarity[25], following by chimera and singletons removed by UCHIME [26]. The next step was assigning taxonomy using the BLAST algorithm with Greengenes 16S rRNA reference database (http://greengenes.lbl.gov). Then, the taxonomic identification was done and various alpha diversity indices were obtained. Moreover, we picked the OTUs of archaea and bacteria from all the OTUs to analyze again following the steps provided above to get accurate abundance of each taxonomic level. The sequencing data as a BioProject were submitted to NCBI SRA database and have been assigned the accession number PRJNA277617.

### Statistical analysis

The unpaired t-test technique was used to evaluate effects of the vaccination treatment on enteric methane emissions, antibody titres in saliva, rumen liquid and serum, abundance of each taxonomic level of Euryarchaeota phylum and abundance of main phylum and taxa. The statistical package used was SPSS (version 18.0). Data were presented as means ± SD, and significance level was set at P<0.05. The QIIME pipeline (version 1.8.0) was also used to an-alyze date of sequencing.

## Results

### Expression of recombinant protein EhaF and purification

**Fig 1:**
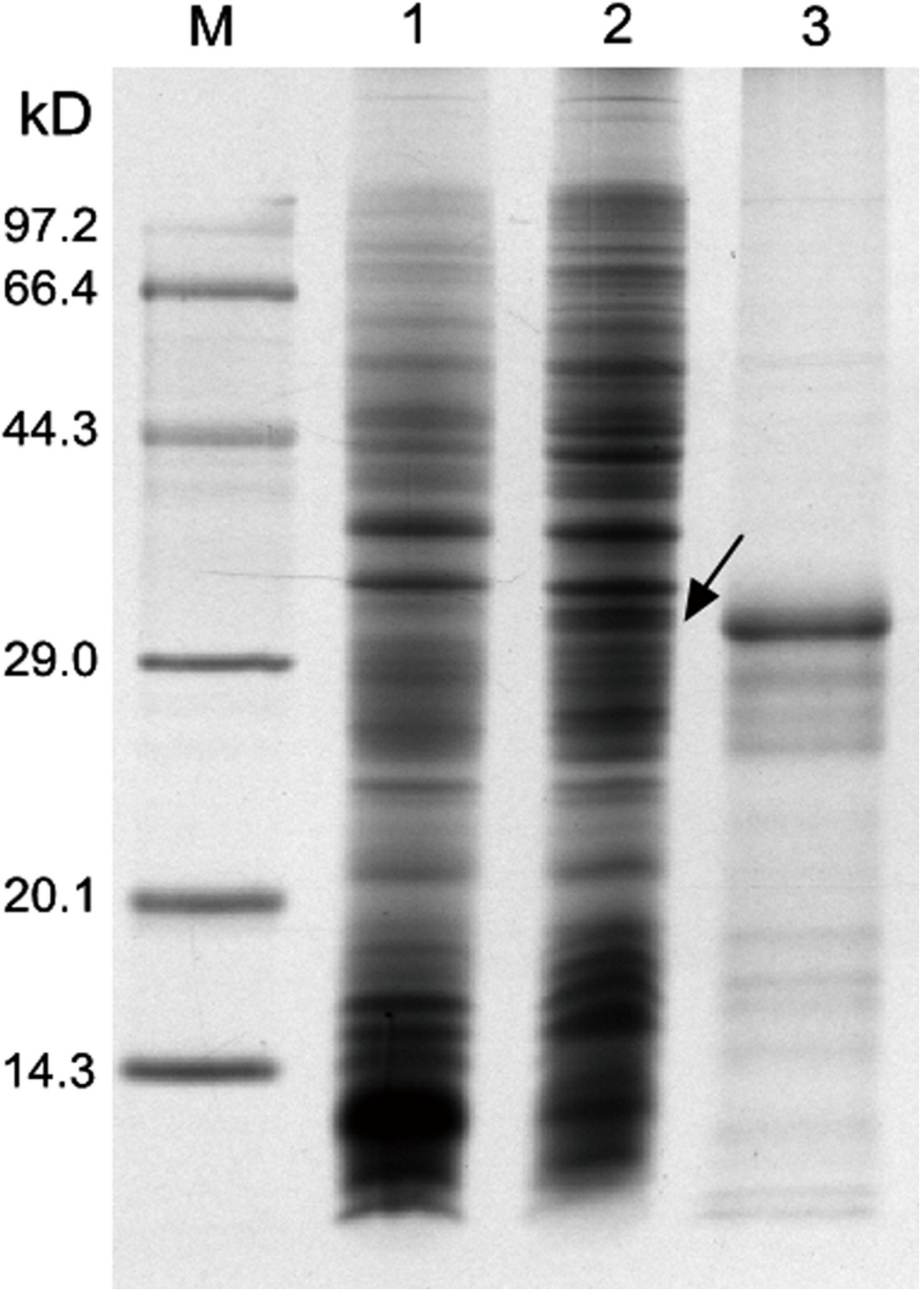
Prokaryotic expression analysis of the recombinant protein EhaF on SDS-PAGE. The recombinant protein was induced with 0.2% ɑ-lactose at 30°C for 16 h. Lanes 1: induced whole-cell harboring pET 30a (+) lysate; Lanes 2: induced whole-cell harboring pET 30a (+)-mru1407 lysate; Lanes3: purified protein after concentrated. The arrowhead points out the position of target protein.

The mru 1407 amplified from DNA of rumen contents by PCR was 579 bp encoding 198 amino acids. The recombinant expression vector pET30a(+)-mru1407 was constructed by ligating the PCR products with *Bam* HI and *Hin*d III restriction sites to corresponding sites of pET30a(+). The recombinant protein EhaF was expected with a molecular weight about 27.5 kDa (with His-tag, S-Tag and enterokinase). It was expressed and no similar size protein was observed in the induced *E*.*coli* transformed with the empty vector pET-30a (+) (Fig 1 lanes 1 and lanes 2). The band of recombinant EhaF has a molecular weight of ~32 kDa according to the protein molecular mass standards. The higher MV than expected might be caused by mobility retardation of His-Tag [27].

The recombinant protein EhaF was purified by Ni-NTA affinity chromatography. The Elution Buffer was used to elute the bound proteins, then the eluate was concentrated fourfold. The ultimate concentration of recombinant protein (Fig 1 lanes 3) analyzed by Bradford method was 1.2 mg/ml.

### Identified by mass spectrometry analysis

**Fig 2:**
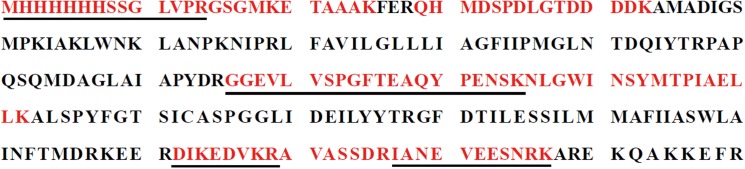
Mass spectrometry identification of recombinant protein EhaF. There were eight matched peptides (Shown in red) and four of them were matched with expected sequence in MS/MS analysis, which were in line.

The purified protein was digested with trypsin and the peptide fragments were analysed by MALDI TOF MS to get Peptide Mass Fingerprinting (PMF) (S1 Fig). The result showed a clear match between recombinant protein EhaF and predicted peptides, which had a Mascot score 310. There were eight matched peptides corresponding to the intensive peak, covering 41% of the recombinant protein sequence (shown in red in Fig 2). Four peptide sequences underlined were matched with predicted protein EhaF sequence. This showed that the recombinant protein was expressed and purified successfully.

### Antibody titer analysis by ELISA

The antibody titres were defined as the reciprocal of the dilution that gave half the maximal optical density (Table 1). Results showed that the mean titer of serum and saliva in the vaccination group of goats were 320000 and 440, respectively, and were significantly higher than those in the control group (P<0.01). The treatment goats also had a significantly higher IgG titres in the rumen fluid (P<0.05).

**Table 1:** Effects of the vaccination treatment on IgG titres against recombinant protein EhaF (mean ± SD) in goats (*n* = 12). The titres were defined as the reciprocal of the dilution that gave half the maximal optical density.

	Antibody titre		
Sample	Control	Vaccination	*P* values
Serum	1833.33±408.25	320000.00±156767.34	0.0040
Saliva	9.33±3.27	448.00±156.8	0.0010
Rumen fluid	1.00±0.00	5.33±3.01	0.017

### Methane production

**Fig 3:**
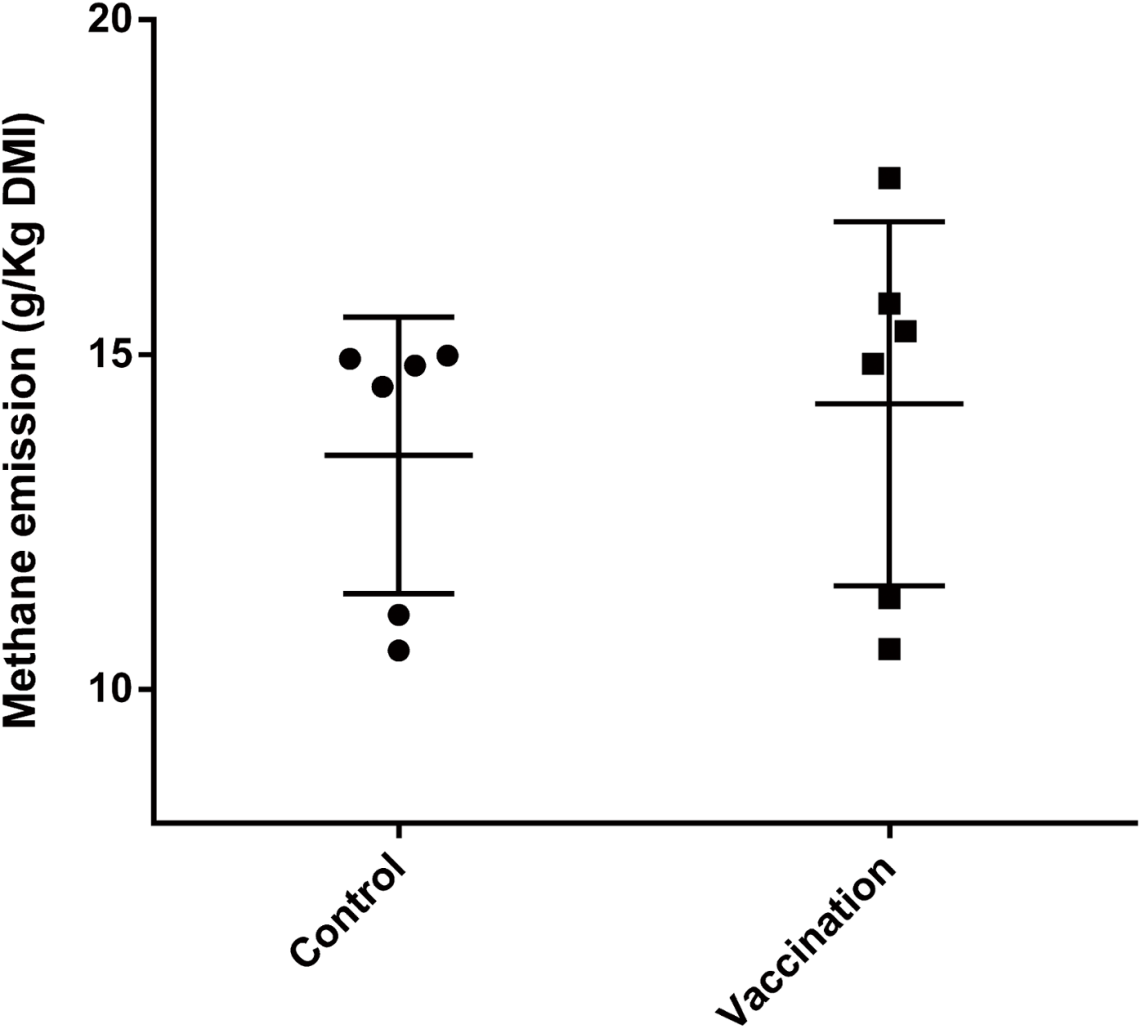
Methane production of goats. Symbols are methane emission of each goat in control (●) and vaccination (■) group. The error bars represent standard deviation of the means.

The CH_4_ emissions for each goat are presented in Fig 3. There was no significant difference in CH_4_ emissions between two groups of goats (P>0.05). The mean CH_4_ emissions in the control and vaccination groups were 13.5 and 14.3g per kg DM intake, respectively.

### Diversity and composition of rumen microbiota

**Fig 4:**
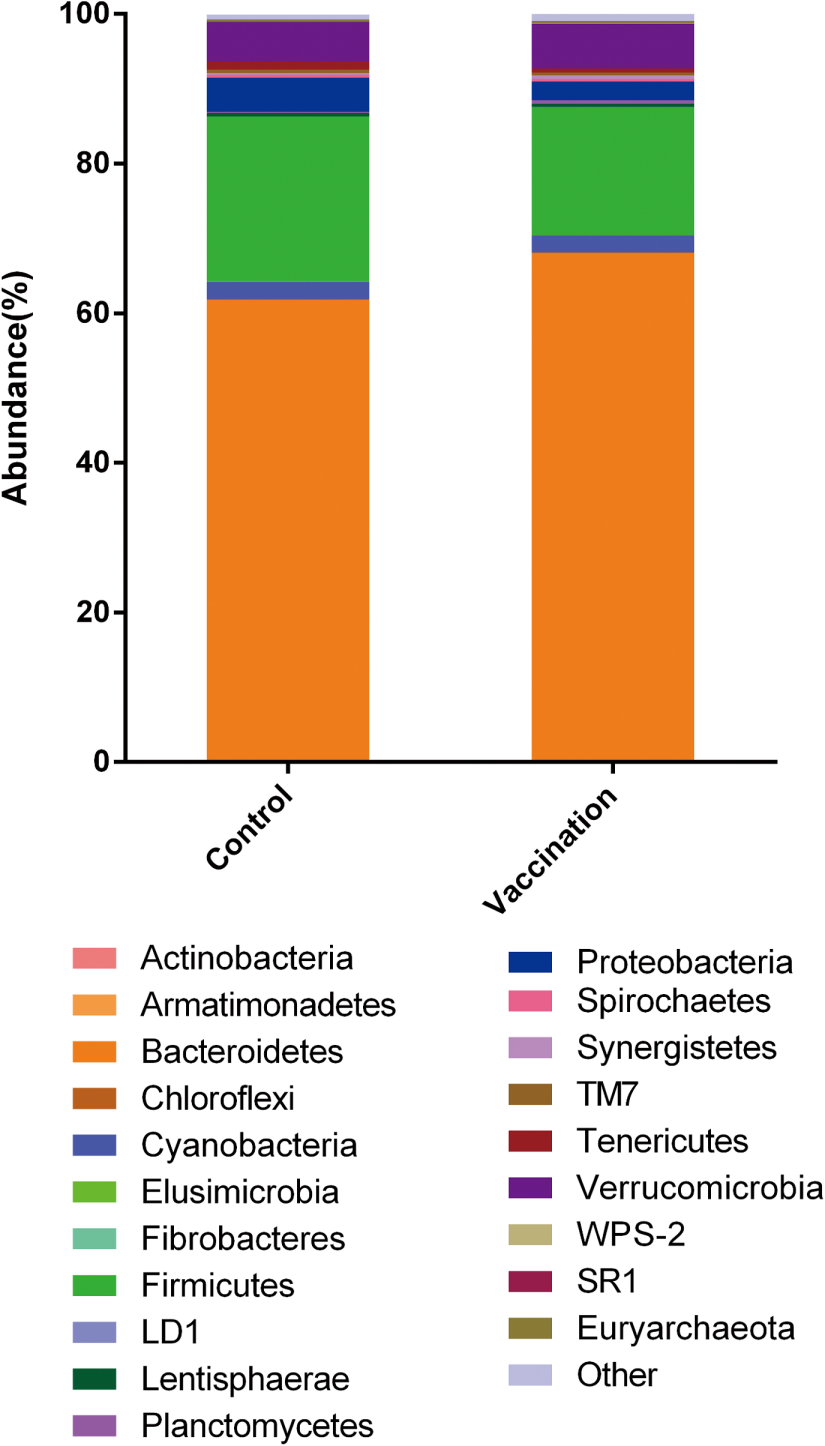
The relative abundance of the phyla of microbes in each group. Each colour represents one phylum and the length indict the mean percentage of each phylum among the whole microbes.

After pyrosequencing of 12 rumen samples using MiSeq PE250 system, 234167 high-quality sequences were obtained (average 19514 sequences per sample). The UCULST assigned these sequences into 4900 OTUs at 97% similarity, with 2776 OTUs belong to Control and 3144 OTUs belong to Vaccination, and there was no marked difference between two groups. The rarefaction curve of OTUs is shown in S2 Fig. Of all the OTUs, 98.9% belongs to bacteria, 0.3% to archaea and 0.8% to unclassified. In total, 359 different taxa were identified from the OTUs, comprising 21 phyla, 36 classes, 62 orders, 96 families and 144 genera. The composition and relative abundance of each phylum in each group are shown in Fig 4. The mean indices of alpha diversity of microbes of each group are presented in Table 2 and these measures have no significant difference between two groups (P>0.05).

**Table 2:** The indices of alpha diversity (mean ± SD) of microbes in each treatment group of goats (*n* = 12).

	PD_whole_tree	chao1	observed_species	shannon
Control	116.43±8.74	2570.64±278.94	1445.45±188.74	7.39±0.47
Vaccination	121.67±12.71	2758.74±347.81	1592.85±251.16	7.66±0.35

Of all the OTUs of archaea, only the Euryarchaeota phylum was detected and the mean abundances of each taxonomic level in this phylum are presented in Table 3. We also compared the abundance of each taxonomy using unpaired t-test programs and these measures had no significant difference between two groups (Table 3).

**Table 3:** Effects of the vaccination treatment on abundance of each taxonomic level of Euryarchaeota phylum (mean ± SD) in goats (*n* = 12).

	% of sequences		
	Control	Vaccination	*P* values
Methanobacteria	58.91±21.40	63.1±21.47	0.74
Methanobacteriales	58.91±21.40	63.1±21.47	0.74
Methanobacteriaceae	58.91±21.40	63.1±21.47	0.74
* Methanobacterium*	1.01±1.68	0.00±0.00	0.17
* Methanobrevibacter*	57.9±22.02	63.1±21.47	0.69
Methanomicrobia	3.90±6.18	2.46±3.45	0.63
Methanomicrobiales	0.33±0.80	0.97±1.70	0.42
Methanomicrobiaceae	0.33±0.80	0.97±1.70	0.42
* Methanomicrobium*	0.33±0.80	0.97±1.70	0.42
Methanosarcinales	3.57±5.58	1.49±1.85	0.41
Methanosaetaceae	0.33±0.80	0.25±0.62	0.86
* Methanosaeta*	0.33±0.80	0.25±0.62	0.86
Methanosarcinaceae	3.25±5.03	1.24±1.94	0.38
* Methanimicrococcus*	3.25±5.03	1.24±1.94	0.38
Thermoplasmata	37.19±18.15	33.39±18.84	0.73
Thermoplasmatales	37.19±18.15	33.39±18.84	0.73
Thermoplasmatales_incertae_sedis	26.27±13.94	26.52±17.69	0.98
* Thermogymnomonas*	26.27±13.94	26.52±17.69	0.98
Unclassified	10.93±6.63	5.11±6.13	0.15
Other	0.00±0.00	1.75±4.05	0.31
Unclassified	0.00±0.00	1.05±1.65	0.15

Of all the bacteria, we compared the relative abundance of each phylum and genus taxonomic level using unpaired t-test programs (Table 4). Of all the phyla observed, the vaccination significantly reduced the abundance of Firmicutes and Tenericutes (P < 0.05), although the treatment had no significant effect on the main component of whole bacteria—Bacteroidetes. The results also showed that three genera belonging to these three phyla varied significantly (P < 0.05)—the vaccination increased the abundance of *Prevotella* but decreased the abundance of genus *Clostridium*, and *RF39. We also observed the vaccinated goats had a significantly lower ratio of Firmicutes to Bacteroidetes (0.25 vs. 0.36) (P<0.05) than the control goats.

**Table 4:** Effects of the vaccination treatment on abundance of main phylum and taxa (mean ± SD) in goats (*n* = 12).

	% of sequences		
	Control	Vaccination	*P* values
Bacteroidetes	62.50±7.13	68.79±2.81	
*Prevotella*	31.6±7.34	41.81±2.48	0.009
Firmicutes	22.26±2.53	17.24±2.92	0.010
*Clostridium*	0.32±0.16	0.15±0.08	0.049
Tenericutes	1.08±0.47	0.56±0.21	0.042
* *RF39*	0.84±0.42	0.34±0.12	0.029

*Taxa that could not be assigned a genus were displayed using the highest taxonomic level that could be assigned to them.

## Discussion

Developing vaccines against methanogens in the rumen of ruminant animals offers an attractive mitigation approach to reduce enteric CH_4_ emissions from ruminant animals, due to its low cost, prolonged efficacy and little side effect [28]. The bioinformation techniques can provide information to identify potential vaccine targets in theory [17] and one of which, GT2, as a vaccination given to cattle, could produce stable salvia antibody in rumen [29]. The EhaF, which is another potential vaccine target, plays an essential anaplerotic role in hydrogenotrophic methanogenesis and is essential for growth of methanogens[18]. However, its efficacy has not been evaluated *in vivo*. Therefore, our study was designed to address this knowledge gap by evaluation of the *in vivo* efficacy of EhaF on enteric CH_4_ emissions and diversity and abundance of microbes in rumen of goats.

The first task of our study was to develop the recombinant protein EhaF. We amplified the gene mru 1407 encoding protein EhaF using fresh rumen fluid samples of mature goats and successfully expressed a new recombinant protein (EhaF) in *E*.*coli* Rosetta. The effect of this new product on the rumen methanogenesis was evaluated *in vivo* using 12 mature goats. Antibodies against EhaF were detected in serum and saliva in the control group, which may be induced by natural exposed methanogens [10]. The vaccinated goats had much higher IgG titres in serum and saliva than the control goats. These indicate EhaF possesses good antigenicity. However, with the vaccinated goats, the titres in rumen fluid were much lower than in saliva. This result was not in accordance with that of [12] and [30], who observed a quarter of and almost same titres in rumen fluid compared with saliva. The difference might be derived from variations in the time of collecting samples. Our samples were collected in the morning before feeding. This indicates that rumen samples we collected might contain less saliva than those collected after feeding. In addition, antibodies from saliva could remain active for only about 8 h in the rumen [31]. However, the present study found that the vaccination significantly increased IgG titres in samples of serum, saliva and rumen. This demonstrated the protein EhaF used in the present study caused significant immune responses in goats and the antibody existed in the rumen.

However, our vaccination treatment did not reduce enteric CH_4_ emissions from goats offered a mixed diet containing concentrates and forage. This result agreed with the finding of a previous study [12], which reported that the vaccine based on five methanogens had no significant effect on enteric CH_4_ emissions, but it altered the composition of methanogens. The vaccination treatment in the present study had no effects on the abundance of any taxon of archaea and index of alpha diversity, indicating that antibodies induced by the vaccination did not effectively change the diversity of microbes and composition of taxa of methanogens we detected. However, the results of the abundance of bacteria showed significant differences between the vaccinated and control groups of goats, especially of the main phylum Firmicutes and genus *Prevotella*. In theory, our vaccine could not alter the composition of bacteria significantly, because EhaF is conserved solely to methanogens [17] and the antibody against recombinant protein EhaF could not target bacteria. Therefore, it is implicated that our vaccine might not be pure recombinant EhaF, but consisted of some unknown peptide which might belong to *E*.*coli*. In the lane 3 of Fig 1, there was more than one band present and that unknown bands were most likely to be residual *E*.*coli* proteins after purification. We speculate that the antibody against these unknown peptides would inhibit *E*.*coli* and the other bacterial communities (Firmicutes and *Prevotella*) might be influenced indirectly, due to the interaction between bacteria in rumen. Moreover, the result also revealed the salvia antibody generated by our vaccine could work in rumen of goats, even though rumen methanogens might not be its target. The present result suggests that the salvia antibody against methanogen protein has potential to work in rumen of goats and there is some mitigation potential to reduce enteric methane emission from ruminant animals by developing vaccines of recombinant methanogens protein against rumen methanogens.

In conclusion, our vaccine, recombinant protein EhaF, induced strong immune responses in goats, but had no significant effect on enteric CH_4_ emissions and methanogen population detected in the present study. These results suggest EhaF was not an effective vaccine target against methanogens for mitigation of enteric methane emissions, using the vaccination procedures described in the study. Nevertheless, the vaccine changed the composition of bacteria, especially the abundance of main phylum Firmicutes and genus *Prevotella*. These results indicated that the vaccine developed in the present study might have potential to influence the ecosystem.

## Supporting Information

S1 ARRIVE Checklist (PDF)

S1 Fig PMF of recombinant protein EhaF. (TIF)

S2 Fig The rarefaction cure of OTUs. Each colour represent one sample and C1-6 and V1-6 denotes control and vaccination group.
(TIF)
