# Supplementary figures and images for: Immunization against Rumen Methanogenesis by Vaccination with a New Recombinant Protein
Source: PLoS One. 2015 Oct 7;10(10):e0140086. doi: 10.1371/journal.pone.0140086 (PMC4596829; doi:10.1371/journal.pone.0140086)

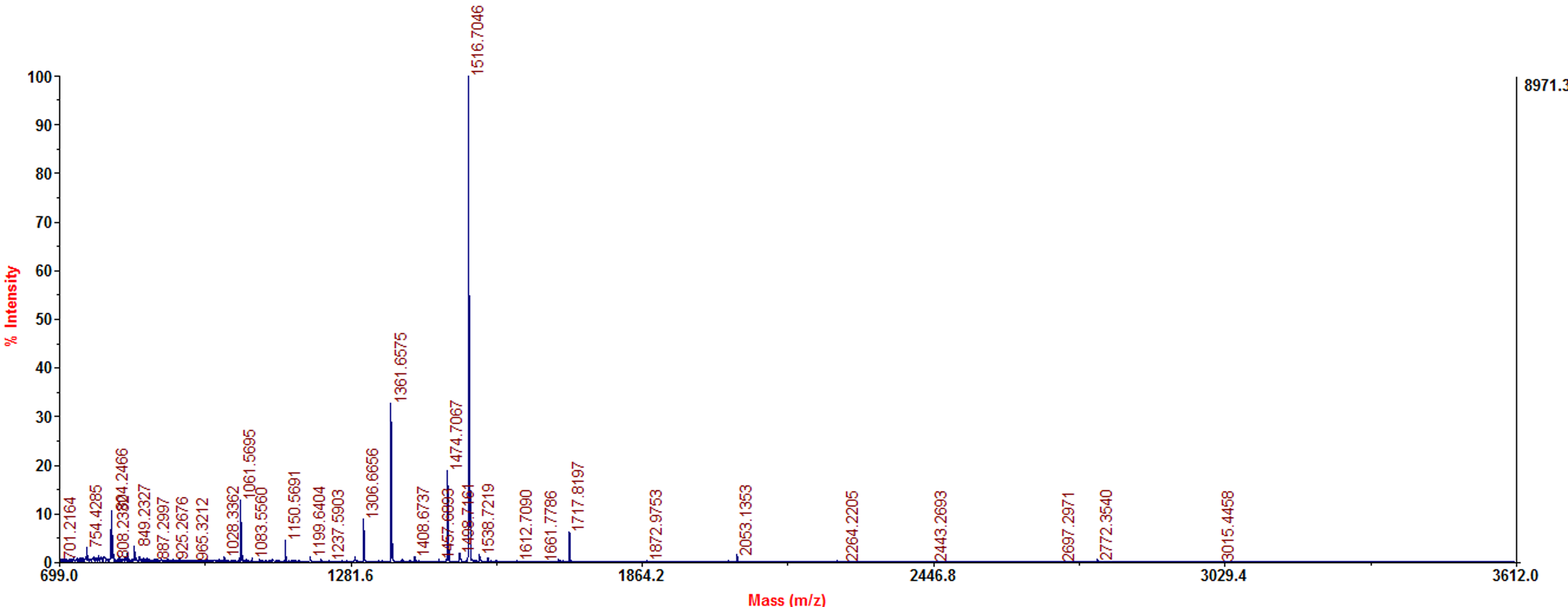

Supplement: S1 Fig — (TIF) [file pone.0140086.s002.tif]
